# MoDHX35, a DEAH-Box Protein, Is Required for Appressoria Formation and Full Virulence of the Rice Blast Fungus, *Magnaporthe oryzae*

**DOI:** 10.3390/ijms23169015

**Published:** 2022-08-12

**Authors:** Shumin Ying, Zhen Zhang, Yanan Zhang, Zhongna Hao, Rongyao Chai, Haiping Qiu, Yanli Wang, Xueming Zhu, Jiaoyu Wang, Guochang Sun, Fucheng Lin

**Affiliations:** 1 State Key Laboratory for Managing Biotic and Chemical Threats to the Quality and Safety of Agro-Products, Institute of Plant Protection and Microbiology, Zhejiang Academy of Agricultural Sciences, Hangzhou 310021, China; 2 College of Chemistry and Life Sciences, Zhejiang Normal University, Jinhua 310029, China

**Keywords:** *Magnaporthe oryzae*, *MoDHX35*, pathogenicity, DExD/H-box protein

## Abstract

The DExD/H-box protein family encompasses a large number of RNA helicases that are involved in RNA metabolism and a variety of physiological functions in different species. However, there is limited knowledge of whether DExD/H-box proteins play a role in the pathogenicity of plant fungal pathogens. In the present work, the DExD/H-box protein MoDHX35, which belongs to the DEAH subfamily, was shown to be crucial in appressoria formation and full virulence of the rice blast fungus, *Magnaporthe oryzae*. The predicted protein sequence of MoDHX35 had typical DEAH-box domains, showed 47% identity to DHX35 in *Homo* species, but had no orthologs in *Saccharomyces cerevisiae*. Deletion of the *MoDHX35* gene resulted in reduced tolerance of the mutants to doxorubicin, a nucleic acid synthesis disturbing agent, suggesting the involvement of *MoDHX35* in RNA metabolism. *MoDHX35*-deleted mutants exhibited normal vegetative growth, conidia generation and conidial germination, but showed a reduced appressorium formation rate and attenuated virulence. Our work demonstrates the involvement of DEAH-box protein functions in the pathogenicity of plant fungal pathogens.

## 1. Introduction

*Magnaporthe oryzae*, a well-known filamentous fungus, causes rice blast, the most devastating worldwide rice disease [1,2]. The fungus can cause systemic symptoms by infecting rice leaves, sheaths, necks, and even rots [3]. In addition to rice, the pathogen may infect a variety of domesticated grasses, including barley, wheat, pearl millet and turf-grass [4]. The infection cycle of this fungus is initiated from a three-cell conidium [5]. Abundant conidia are produced on the surface of a lesion and repeat the reinfection during the rice growing season, surviving the winter to start a new infection cycle in the following year. The conidium exudes mucilage that helps it stick to the surface of the host to aid germination within a few hours in the right environment [6]. Once germinated, the germ tube develops a specialized infection structure called the appressorium on its tip. The appressorium possesses a thick cell wall and accumulates highly concentrated glycerol to generate enormous turgor [7,8]. Relying on the turgor, a thin penetration peg emerges under the mature appressorium to puncture the host surface, enter a plant epidermal cell, and commence the invasive development. The capability to generate appressoria is thus of key importance for the pathogenicity of the rice blast fungus.

The signal pathways that control appressorial morphogenesis in *M. oryzae* were extensively investigated in recent decades [9,10]. *MPG1*, a tiny hydrophobin-encoding gene, is thought to be involved in signal sensing on the host surface [11,12,13,14]. Pathogenicity was greatly reduced when *MPG1* was deleted in *M. oryzae*. *PTH11*, which encodes a transmembrane protein, acts as an upstream effector of appressorium differentiation in response to surface stimulation [15,16]. Due to a deficiency in appressorium development, the *PTH11-*deleted mutants are nonpathogenic. In appressoria, the cAMP-mediated *CPKA* (catalytic subunit of protein kinase A) pathway is important in host surface recognition and turgor generation [17,18,19]. The *MAC1* gene, which encodes adenylate cyclase, is necessary for vegetative growth, conidia generation, conidial germination and appressorium formation [20]. The *PMK1* mitogen-activated protein kinase pathway is also involved in appressorium formation [9,21]. The *PMK1*-deleted mutants do not generate appressoria and, hence, are nonpathogenic. MST12, a potential transcription factor, functions downstream of *PMK1* to regulate genes involved in appressorial penetration and infectious growth [21,22]. The *MST12*-deleted mutant produces melanized appressoria with normal appressorial turgor but no penetration pegs, probably due to cytoskeleton abnormalities in the mature appressorium [23]. G protein pathway, Ca^2+^ signaling pathway, Rac pathway and regulators of G protein signaling (Rgs) were all shown to be linked to appressorial formation and pathogenicity [24,25,26,27]. Transduction pathways give the signals to the nucleus, activating structural and metabolomic genes. Multiple genes and proteins that control DNA duplication, gene transcription, and protein expression are required for these activities.

Helicases, which use the energy from ATP hydrolysis to unwind double-stranded nucleic acid into single strands, are essential regulators of gene expression in all living organisms [28]. Almost all of the RNA metabolic processes, such as translation, transcription, ribosome biogenesis, RNA splicing, RNA transport, RNA editing and RNA degradation are facilitated by the helicases [28,29]. The DExD/H-box family, is a helicase protein family which takes its name from the four conserved amino acids of motif II (which is equivalent to the Walker B motif of ATPases) [30]. Members of the DExD/H-box family are involved in translation initiation, nuclear and mitochondrial mRNA splicing, nuclear export, and ribosome biogenesis [31,32,33,34,35,36]. The DExD/H-box family has a basic structure that includes a nip between two domains with an RNA binding site on each side [32]. Because of their basic structure, DExD/H-box proteins can use ATP to unwind RNA–RNA duplexes, RNA–DNA duplexes, or RNA–Protein complexes. In mammalians and *Saccharomyces* *cerevisiae*, several DExD/H-box members were identified and described. However, little is known about the roles of DExD/H-box family members in plant fungal pathogens. To reveal whether the DExD/H-box proteins are involved in the pathogenicity of the plant pathogenic fungi, in the present work, we functionally characterized MGG_02518, a predicted gene encoding a protein belonging to the DEAH-box subfamily.

We previously isolated a random T-DNA inserted mutant, S32, which showed an attenuated virulence in rice. By identifying the T-DNA insertion site in S32, the promoter region of MGG_02518 was found to be disrupted, implying that MGG_02518 may play a role in the pathogenicity of the fungus. MGG_02518 encodes a DEAH-box protein that is most similar to *DHX35* in *Homo species*; hence, it was assigned as *MoDHX35* (*M. oryzae DHX35*) in this work. By using the gene replacement strategy, we confirmed the roles of *MoDHX35*. Tolerance to the nucleic acid synthesis disturbing agent was reduced when *MoDHX35* was deleted, implying that *MoDHX35* plays a function in RNA metabolism. The *MoDHX35* null mutant exhibited a reduced ability to generate appressoria and attenuated virulence, but had no effect on vegetative growth, conidia genesis, or conidial germination. These data demonstrate a case of a member of the DExD/H-box protein family being involved in the pathogenicity of rice blast fungus.

## 2. Results

### 2.1. Isolation and Analysis of the Gene Locus Inserted by T-DNA

The T-DNA insertion mutant of *M. oryzae*, S32, that we had previously constructed, reduced the pathogenicity on rice. Retrieving the T-DNA insertion site in S32 revealed that the disruption of MGG_02518 is likely the reason for its infection defects. To further confirm the sequence and coding information of MGG_02518, the full-length cDNA was isolated from mycelia of the *M. oryzae* strain Guy11 by RT-PCR and sequenced. A comparison with the sequence in the genome database showed that the deduced CDS and peptide of MGG_02518 were completely identical. The MGG_02518 ORF is 2030 bp containing a 138 bp intron and encodes a predicted protein of 672 amino acids.

**Figure 1:**
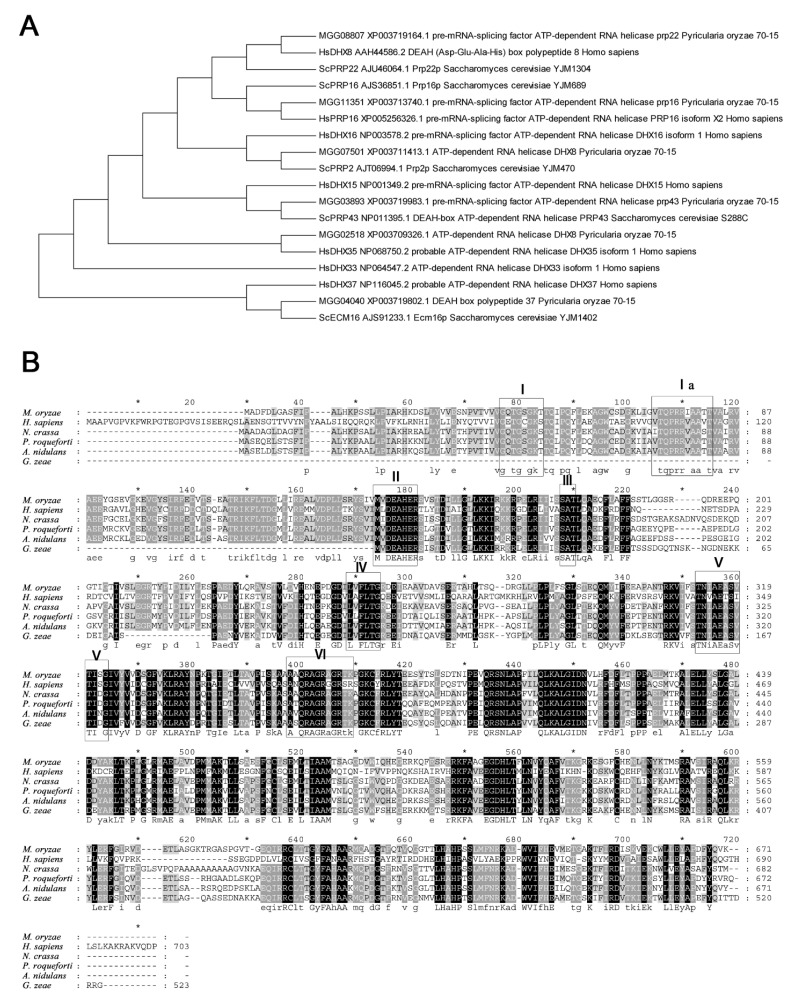
Similarity of *MoDHX35* homologs. (**A**) Phylogenetic relationship of *MoDHX35* (MGG_02518) and its closely related proteins in *Magnaporthe oryzae*, *Saccharomyces cerevisiae* and *Homo species*, calculated with neighbor-joining method using the MEGA 5.0 program. (**B**) Amino acid sequences of *HsDHX35* (NP_068750.2) from humans, *NcDHX35* (XP_963507.1) from *Neurospora crassa*, *PrDHX35* (XP_038929158.1) from *Penicillium roqueforti*, *AnDHX35* (EHA17759.1) from *Aspergillus nidulans* and *GzDHX35* (XP_381721.1) from *Gibberella zeae* (*Fusarium graminearum*) were aligned using Clustal W. The identical amino acids are highlighted against a black background, conserved residues are shown on a dark gray background, and similar residues on a light gray background.

**Figure 2:**
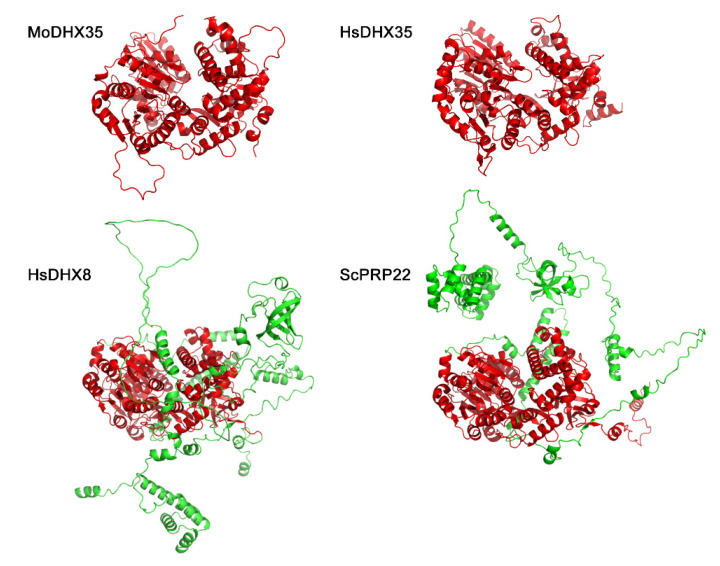
Three-dimensional structures of *MoDHX35*, *HsDHX35*, *HsDHX8* and *ScPRP22* generated by the structure prediction server AlphaFold2 database (https://www.cloudam.cn/v2/console/create-job, accessed on 1 December 2021). The conserved portions of the proteins are in red.

By BlastP searching against the NCBI database, MGG_02518 was found to be, among the annotated proteins, most similar to *DHX35*, a DEAH-box protein in *Homo species*, with 47% identities at the peptide level. The first hit in *S. cerevisiae* to MGG_02518 was Prp22, which is an ortholog to *H. species DHX8*. However, MGG_08807 is the protein in *M. oryzae* most similar to *S. cerevisiae* Prp22 (Figure 1A). The seven representative motifs in DExH-box proteins were detected in MGG_02518. MGG_02518 was thus assigned as a *DHX35* ortholog in *M. oryzae* and named *MoDHX35* in this work. Although no ortholog was found in *S. cerevisiae*, the homologous proteins to *MoDHX35* were detected in the genomes of the closely related fungal species. Most of these proteins are highly conserved and share more than 60% of their identity with *MoDHX35* (Figure 1B). Three-dimensional structures generated by the structure prediction server AlphaFold2 database also demonstrated the resemblance between *MoDHX35* and *HsDHX35* (Figure 2).

### 2.2. MoDHX35 Is Up-Regulated during Appressoria Formation

**Figure 3:**
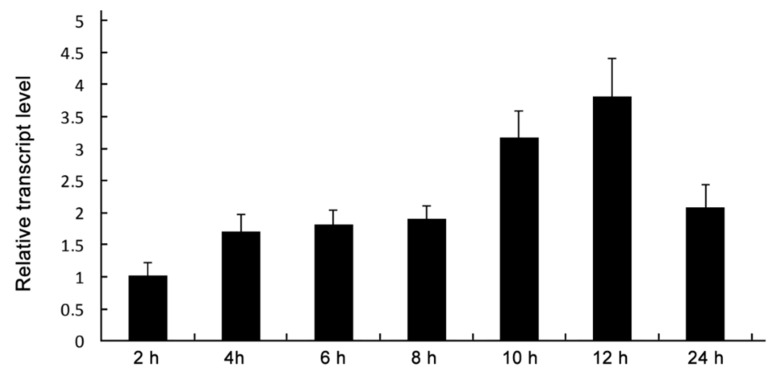
Sequential expression of *MoDXH35*. Relative transcript abundance of *MoDXH35* during conidial germination and appressorial formation. The transcript abundance normalized to β-tubulin gene (MGG_00604) was measured by quantitative RT-PCR at time points and compared to that in the non-incubated conidia.

To assess the expression profile of *MoDHX35*, the conidia suspension was allowed to germinate and form appressoria on plastic slices, and the relative transcription levels of the gene were tested using quantitative RT-PCR. *MoDHX35* was found up-regulated gradually during germination and appressoria formation, reaching the peak value at 10 to 12 h for induction, the key period of appressorium formation (Figure 3).

### 2.3. Gene Replacement of MoDHX35 and Mutant Recovery

**Figure 4:**
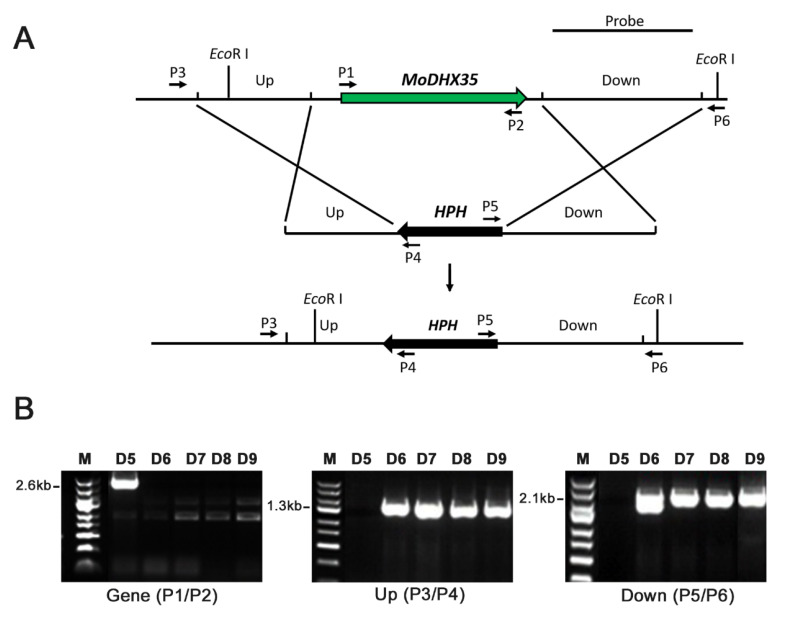

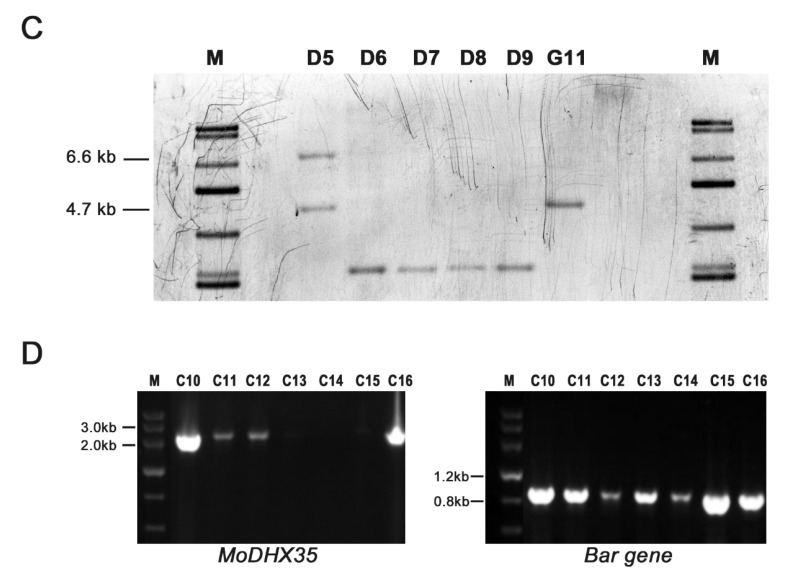
*MoDHX35* gene deletion and mutant complementation. (**A**) Diagram showing that the *MoDHX35* coding region was replaced by *HPH* cassette. The probe marked downstream *MoDHX35* was used for Southern blotting. The primers marked for mutant selection were as follows: P1, *MoDHX35*-Genecheck-F1; P2, *MoDHX35*-Genecheck-R1; P3, *MoDHX35*-Upcheck-F1; P4, Sequence-Up; P5, Sequence-Down; P6, *MoDHX35*-Downcheck-R1. (**B**) Genomic PCR was used to validate the deletion of *MoDXH35*. The *MoDXH35*, upstream fragment and downstream fragment were amplified, respectively, with the primer pairs P1/P2, P3/P4, P5/P6. (**C**) Total genomic DNAs were isolated from the wild type (G11), ectopic transformant (D5), and potential Δ*MoDHX35* mutants (D6, D7, D8 and D9), digested with *Eco* RI and subjected to Southern blotting. The 6.6-kb hybridization band was detected in the wild type, whereas the 4.7-bp bands were present in the four potential mutants, consistent with the gene deletion events. Ectopic transformant generated two bands, one of which was equal in size to the wild type. (**D**) Genomic PCR was used to validate the complement transformants of *MoDXH35* by amplifying the fragment *MoDXH35* and *Bar* gene.

The gene placement vector P1300-HPH-*DHX35*KO was transferred into Guy11 via *At*MT (Figure 4A). One hundred and sixty-three hygromycin B-resistant transformants were obtained. Twenty-two transformants, selected randomly, were single-spore isolated and then screened preliminarily by PCR (Figure 4B). Four transformants (*DHX35-6*, *DHX35-7*, *DHX35-8*, and *DHX35-9*) lacking the amplicon of the *MoDHX35* locus were identified as potential mutants and were further confirmed by Southern-blotting. Their genomic DNA was digested with *Eco* RI and hybridized with a 2127 bp probe downstream *MoDHX35*. The null mutants with gene replacement occurrence produced a 4.7 kb hybridized band, in contrast to the wild-type Guy11, which produced a 6.6 kb band. The random insertion transformants possessed a 6.6 kb band, representing the wild-type *MoDHX35* locus, and another band in random sizes (Figure 4C). Thus, *DHX35-6*, *DHX35-7*, *DHX35-8* and *DHX35-9* are regarded as the true *MoDHX35* null mutants, and *DHX35-5* is a random insertion transformant. Reverse transcription PCR was used for further confirmation and showed that the *MoDHX35* transcription was eliminated in the mutants. The four mutants were identical in colonial morphology, radical growth and conidiation; *DHX35-9* was used as a representative in this study. The complement plasmid p1300-BAR-HB-*MoDHX35* was reintroduced into the *DHX35-9* genome. The complemented transformants, *DHX35-9-10* and *DHX35-9-16*, were confirmed by genomic PCR and RT-PCR. *MoDHX35* in *DHX35-9-10* and *DHX35-9-16* were transcribed at a comparable level to that in wild-type strain Guy11 (Figure 4D).

### 2.4. Loss of MoDHX35 Increases the Sensitivity of the Mutant to Doxorubicin

**Figure 5:**
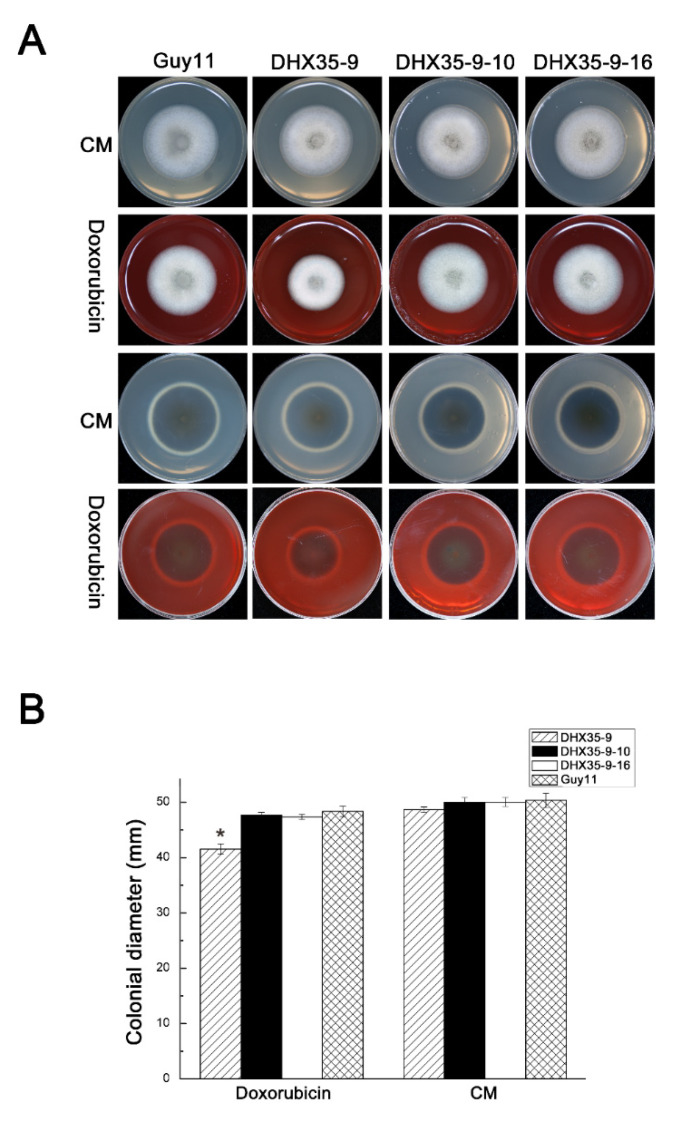
*MoDHX35* mutant is hypersensitive to doxorubicin. (**A**) The strains were cultured on CM supplemented with 75 μg/mL doxorubicin for 6 d. (**B**) The colonial diameters were measured and the relative inhibition rates compared. Standard deviations are indicated by the error bars. The asterisks indicate significant differences at *p* < 0.05 level.

Doxorubicin, which is usually used to cure cancer, can block the synthesis of nucleic acid. Here the null mutant, complemented transformants and wild-type Guy11 were grown on CM plates with 75 μg/mL doxorubicin. The pictures show clearly that the null mutant growth rate is slower than that of Guy11 (Figure 5A,B). However, the complemented mutants grow at almost the same rate as that of the wild type. These results indicate that *MoDHX35* may play an important role in nucleic acid synthesis.

### 2.5. MoDHX35 Contributes to M. oryzae Appressorium Formation 

To determine which infection steps resulted in the pathogenicity defects of *MoDHX35* mutant, germination and appressoria formation of the null mutant were compared to those of the wild type and the complement strains. At 2 h post incubation, 97% Guy11 conidia germinated, as did 95.2% of *DHX35-9* conidia, without obvious differences between them, indicating *MoDHX35* has no effect on *M. oryzae* conidial germination.

**Figure 6:**
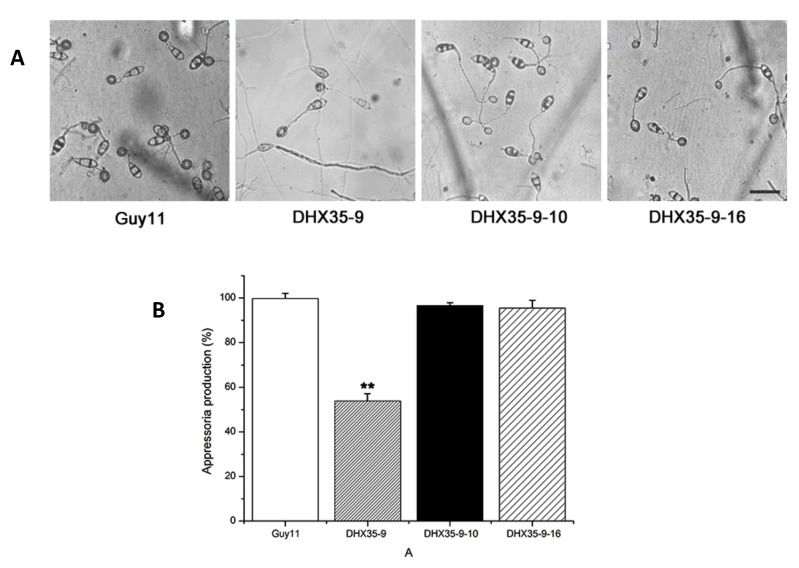
*MoDHX35* is required in appressorial development. (**A**) Microscopy of the appressoria formed at 24 h post incubation on hydrophobic surface of the wild type, *MoDHX35*-deleted mutant, and complement strains. The mutant was significantly reduced in its ability to form appressoria, and the appressoria of the mutant were less pigmented than those of the control strains. The bar = 20 µm (**B**) The appressorial formation rates of *MoDHX35*-deleted mutant, wild-type and complemented strains were calculated. The double asterisks indicate significant differences at *p* < 0.01 level.

All tested strains were able to form an appressorium (Figure 6A). However, as shown in Figure 6B, only 53.82% conidia (*n* = 1000) of the null mutants produced appressoria, compared with 99.74% Guy11 conidia. This comparison statistic shows that the knockout of *MoDHX35* really affected the ability to form appressoria. Appressoria formation of the complement mutants was restored, which means that the appressorium formation reduction in the null mutant is due to the *MoDHX35* knockout itself.

### 2.6. MoDHX35 Is Required for M. oryzae Pathogenicity 

**Figure 7:**
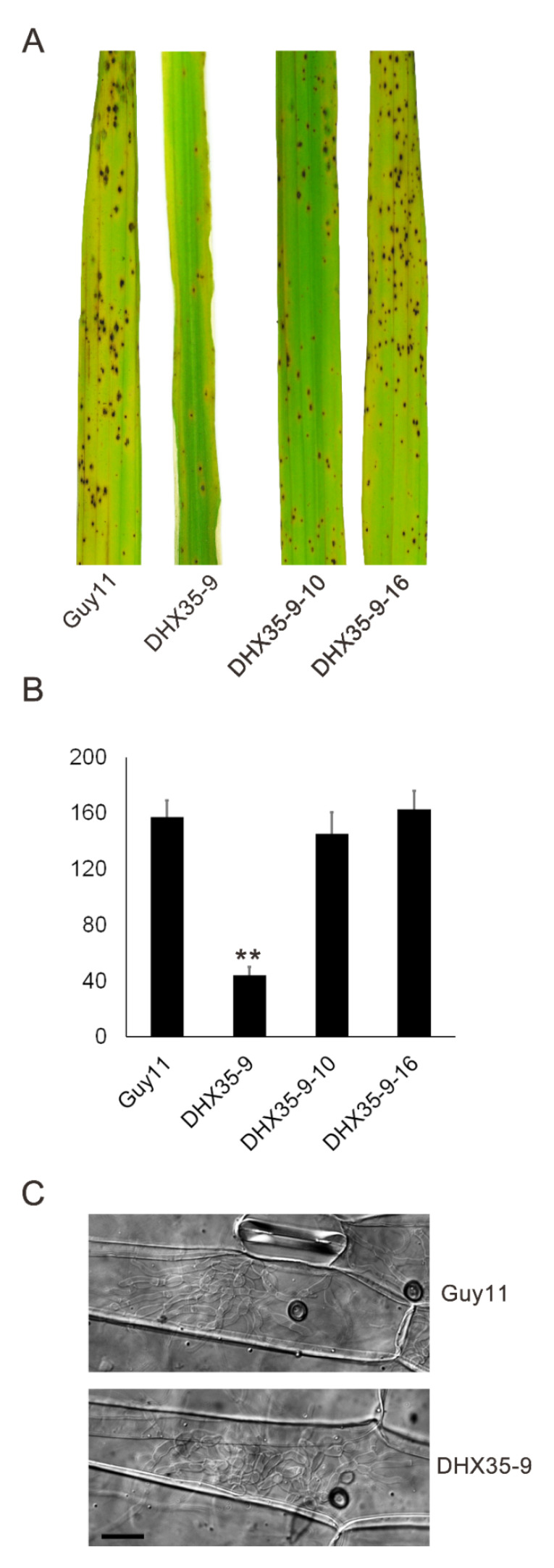
Pathogenicity test of the *MoDXH35*-deleted mutants. (**A**) Spray inoculation with conidial suspension (1 × 10^5^ conidia/mL) on 2-week-old rice cultivar CO39, recorded at 7 days post inoculation (dpi). (**B**) The numbers of lesions on 5-cm leaves were counted and statistically analyzed. Error bars represent the deviation from three replicates and double asterisks indicate significant differences at *p* < 0.01 level. (**C**) The infection structures of the mutant and the wild type were compared on barley leaves. The detached barley leaves were drop inoculated with the conidia suspension and incubated for 36 hpi. The infection hyphae of the mutant were found to develop at an equivalent level as that of the wild type. The bar =10 µm.

A pathogenicity assay was performed on the rice susceptible cultivar CO39. Conidial suspensions from Guy11, *DHX35-9*, *DHX35-9-10* and *DHX35-9-16*, in equal concentrations, were spray inoculated onto rice seedlings. At 7 d post inoculation, the rice leaves inoculated with the wild type formed typical symptoms, while the *DHX35-9* mutant exhibited significantly reduced virulence compared with that of the wild type. An average of 24.0 ± 2.6 lesions was generated on the 5-cm leaves inoculated with *DHX35-9*, significantly lower than the 115.0 ± 15.7 caused by Guy11. On the other hand, the complement strains *DHX35-9-10* and *DHX35-9-16* exhibited comparable virulence to that of the wild type on rice leaves (Figure 7A,B). The data indicate that *MoDHX35* is required for the full virulence of the rice blast fungus on rice. We then examined the infection structures on barley leaves under a microscope. As observed on the artificial surface, the DHX35-9 mutant forms appressoria at a reduced rate on barley leaves. Nevertheless, the appressoria of the mutant has infection capability, and the development of the infection hyphae did not exhibit significant difference to those of the wild type (Figure 7C). The data indicate that the attenuated virulence of the *MoDHX35* null mutant was due, predominantly, to the reduction in appressoria formation.

### 2.7. Deletion of MoDHX35 Does Not Affect the Sexual Development of the Fungus

**Figure 8:**
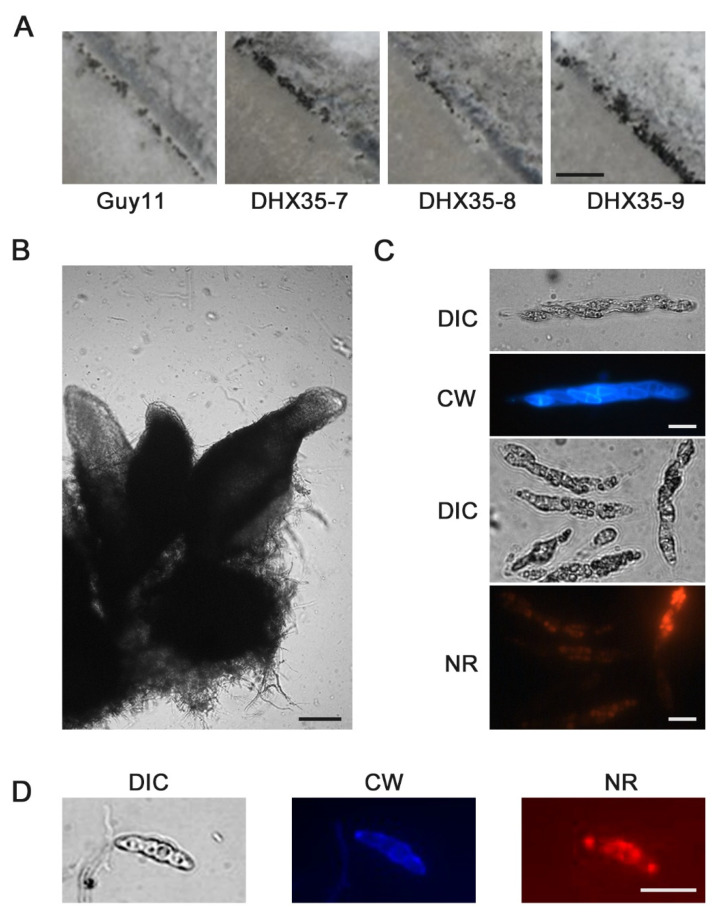
Sexual development of *MoDHX35* mutants is normal. (**A**) The wild type and mutant were cross cultured with 2539 strains on OMA medium for 30 d. The black dotted perithecia was found on the junction zones of the strains. The bar = 1 mm. (**B**) Three typical perithecia. The perithecia have a spherical body with a long tubular beak. The bar = 50 µm. The cell wall of ascus (**C**) and ascospore (**D**) were stained with 0.1% Calcofluor white (CW) into blue fluorescence and the lipids inside the cell were stained with 0.1% Nile-red into red. The bars =10 µm.

After cross-inocubated with 2539 strain for 30 days, both of the mutants and wild-type strain generated typical perithecia on oat medium (OMA) (Figure 8A,B). Calcofluor white and Nile-Red flourescent staining were used to highlight respectively the cell wall and cellular lipid in the asci and the ascospores. With the aid of the Calcofluor white and Nile-Red staining, eight ascospores could be found clearly in a mature ascus of the wild type, as well the *MoDXH35* deleted mutant (Figure 8C,D).

### 2.8. MoDHX35 Mutants Are Not Temperature Sensitive

A number of DExD/H-box mutant genes in *S. cerevisiae* were temperature-sensitive, so we tested the growth rates of the *MoDHX35*-deleted mutants at different temperatures. The results show that the growth rates of all tested strains decrease at the temperatures above or below 28 °C, especially at high temperatures, but no significant difference was found in growth rates between mutants and wild types at the same temperature (Figure S1). This indicates that the *MoDHX35* mutants are not temperature sensitive.

### 2.9. Deletion of MoDHX35 Does Not Alter the Nutritional Utilization, Osmic STRESS or Resistance to Chemicals of the Fungus

We used a culture media with different carbon and nitrogen sources, CM-C (without carbon source), CM-N (without nitrogen source), CM-C + 50 mM sucrose, CMC + 1% Tween80, CM-C + 50 mM sodium acetate, CM-C + 1% oleic acid, CM-C + 1% olive oil and MM, to test whether the deletion of *MoDHX35* influenced the nutrient utilization of the fungus. On the above media, the wild-type Guy11, and the mutants DHX35-6, DHX35-8 and DHX35-9 exhibited no significant differences in growth rates or colonial morphology (Figure S2). On CM containing 0.2 M, 0.4 M, 0.6 M and 0.8 M NaCl, the *MoDHX35* deleted mutants and wild type grew at equivalent rates and showed no significant difference in terms of colonial morphology (Figure S3A). This indicates that *MoDHX35* does not participate in carbon and nitrogen utilization, lipid degradation or endurance of osmic stress.

To compare the cell-wall integrity of the mutants and the wild type, we cultured the strains on CM supplemented with Calcofluor white for 7 days and found no difference between the mutant and wild type (Figure S3B). We also compared the endurance of the mutants and the wild type to Carbendazim, a commonly used fungicide and Cycloheximide, an inhibitor of protein biosynthesis, and did not detect obvious differences either (Figure S3C,D).

## 3. Discussion

In this study we characterized a DExD/H-box protein-coding gene, *MoDHX35* (MGG_02518), which plays a role in the pathogenicity of rice blast fungus. MoDHX35 protein possesses seven typical conserved domains of DExD/H-box proteins and shows 47% similarity to DHX35, a DEAH-Box helicase in *H. species*. The *MoDHX35*-deleted mutants showed more sensitivity to doxorubicin, a nucleic acid synthesis disturbing agent, also suggesting the role of MoDHX35 as an RNA helicase. The deletion of *MoDHX35* affected the formation of appressoria and significantly reduced the virulence on rice leaves. In addition, the deficiency of the *MoDHX35* deleted mutants could be recovered by reintroduction of a *MoDHX35* cassette. The expression of *MoDHX35,* reflected by real time RT-PCR, indicates that *MoDHX35* is up-regulated in 10 to 12 h post induction of the conidia, a key stage for appressorium differentiation (Talbot, 1995; 2003), corresponding well with the phenotype of the mutants and supporting the involvement of *MoDHX35* in appressorium formation. To date, only very limited DExD/H-box proteins were documented in *M. oryzae*, including two dicer-like proteins (DCL), MDL-1 and MDL-2. The gene deletion indicates that *MDL-2* is responsible for the RNA silencing pathway in *M. oryzae* [37]. Meanwhile, the *MDL-2* mutant was also found to have a slightly slower growth rate at 22 and 30 °C compared with the wild type. Nevertheless, whether the DCLs are involved in the fungal pathogenicity had not been reported. Therefore, our data provides a case illuminating the involvement of the DExD/H-box family proteins in the pathogenicity of the plant fungal pathogens.

DExD/H-box family proteins are widely accepted as RNA helicases which are related to various processes of RNA metabolisms and are, thus, involved in several important metabolic processes of life development [38]. We, therefore, also checked the other phenotypes related to pathogenicity and fungal development, including nutritional utilization, osmic stress and resistance to chemicals, but few were found changed, compared to the wild type, except for the sensitivity to doxorubicin. Thus, *MoDHX35* is likely a DEAH protein specifically mediating the pathogenicity in *M. oryzae*, and the deficiency of the mutants in pathogenicity is mainly due to the failure in appressorium formation.

According the BlastP results, the most similar hit to MGG_02518 in *S. cerevisiae* is Prp22. The *S. cerevisiae* Prp22 mediates mRNA release from the spliceosome and unwinds RNA duplexes [39]. In *H. species*, *DHX8* is the homolog to *S. cerevisiae* Prp22. Meanwhile, *DHX35*, as well as *DHX15*, *DHX16*, *DHX37* and *DHX38* are also regarded as the paralogs to *DHX8* for their similarity in sequences and possible related functions in RNA metabolisms [40,41]. The phylogenetic tree made from the closely related DHX and PRP proteins in *M. oryzae*, *S. cerevisiae* and *H. species* support MGG_02518 as the homolog to *DHX35*, and the MGG_08807 is the protein in *M. oryzae* most similar to *S. cerevisiae* Prp22 and *H. species*, *DHX8.* Nevertheless, a number of possible paralogs to *MoDHX35* were present in the *M. oryzae* genome, such as MGG_08807, MGG_03893, MGG_04040, MGG_07501 and MGG_11351. Whether these proteins are truly the paralogs or orthologs of the known DExD/H proteins, and what their exact functions in metabolisms, development and pathogenicity may be in *M. oryzae* are attractive topics and worthy of further investigation. Moreover, we have, in fact, tried to delete all of the possible DExD/H protein-encoding genes using knockout strategies, but failed to obtain the null mutant for most of the genes (data not shown). These facts may reflect the key roles of the DExD/H proteins in fundamental life activities: once deleted, the mutants are lethal.

In summary, in the present work, we demonstrated that, *MoDHX35*, a DEAH-box like protein, is involved in the appressorium formation and pathogenicity of the rice blast fungus, *M. oryzae*.

## 4. Material and Methods

### 4.1. Fungal Strains and Growth Conditions

The strains used in this study are listed in Table 1. *M. oryzae* wild-type strain Guy11, mutants and complementary strains were routinely cultured on complete medium (CM) at 28 °C, with a 16-hour light/8-hour dark cycle. For liquid cultivation, approximately 1 × 10^5^ conidia were placed in 100 mL liquid CM at 28 °C and shaken at 150 rpm for 2 days.

### 4.2. MoDHX35 Isolation and Sequence Analysis 

The CDS fragment of *MoDHX35* was amplified using the primers MoDHX35-CDS-F1 and MoDHX35-CDS-R1 from cDNA samples produced on RNA isolated from mycelia of Guy11 strain. The product was cloned into pEASY-T3 vector (TransGen, Beijing, China) and sequenced. The homologous sequences were retrieved by searching from the NCBI database with the BlastP program. Sequence alignment was processed using the Clustal W module in MEGA5.10, with a gap opening penalty of 10 and gap extension penalty of 0.2. The neighbor-joining method with a bootstrap test of 2000 was used to build the phylogenic tree.

### 4.3. Vector Construction, Gene Deletion and Mutant Complementation

p1300-HPH, a binary plasmid for *MoDHX35* deletion, was created by inserting a 1344-bp hygromycin phosphotransferase (*HPH*) cassette into pCAMBIA1300 as a backbone for gene substitution. Following this, using primers MoDHX35-Up-F1 and MoDHX35-Up-R1, a 1335-bp upstream flanking segment of *MoDHX35* was amplified from Guy11 and inserted into P1300-HPH between the *Sac*I and *Kpn*Ι sites to generate p1300-HPH-MoDHX35UP. Similarly, a 2124-bp downstream flank of *MoDHX35* was amplified with primers MoDHX35-Down-F1 and MoDHX35-Down-R1 and inserted into P1300-HPH-MODHX35UP between the *Bam*HI and *Hin*d III sites to generate the disruption vector p1300-HPH-MoDHX35KO. The p1300-HPH-MoDHX35KO was introduced into Guy11. The resulting Hygromycin B-resistant transformants were screened by PCR using the primer-pairs MoDHX35-Genecheck-F1 and MoDHX35-Genecheck-R1, Hph-Check-F1 and Hph-Check-R1, MoDHX35-Upcheck-F1 and Sequence-Up, and MoDHX35-Downcheck-R1 and Sequence-Down. The transformants with 2.6 kb *MoDHX35* gene negative, 1.3 kb 5′ flank positive and 2.1 kb 3′ flank positive were selected as the potential knockout mutants. The mutants emerging from the gene replacement were confirmed by Southern-blotting analysis and selected for phenotypic study.

To construct the complement plasmid of *MoDHX35*, a 4200 bp fragment containing the full length of the *MoDHX35* gene and 1700 bp upstream of the start codon was amplified by using the primers MoDHX35-Com-F1 and MoDHX35-Com-R1, and cloned into pEASY-T3 (TransGen, Beijing, China). After confirmation by sequencing, the fragment was inserted into P1300-BAR, a plasmid containing the *BAR* gene (Glufosinate ammonium resistance) in the pCAMBIA1300 backbone, to generate the complementary vector p1300-BAR-HB-MoDHX35. The p1300-BAR-HB-MoDHX35 vector was introduced into one of the knockout mutants. The resulting complement transformants were confirmed by PCR with primers MoDHX35-Genecheck-F1 and MoDHX35-Genecheck-R1.

All vectors were integrated into *M. oryzae* strains via *Agrobacterium tumefaciens*-mediated transformation (*At*MT). CM plates containing corresponding antibiotics (250 μg/mL Hygromycin B (Roche, Basel, Switzerland) or 200 μg/mL Glufosinate-ammonium (Sigma, St. Louis, MO, USA) were used to screen the transformants. The primers used in this work are listed in Table 2.

### 4.4. Vegetable Growth and Conidiation on Culture Media

The vegetative growth of all tested strains was examined by inoculating 5 mm discs of mycelia on 9 cm diameter culture media for 9 d and then measuring the colonial diameters. Sensitivity assays were operated the same way on the CM plates supplemented with corresponding agents. To estimate conidiation, the conidia were harvested by washing the 7-day-old colonies on CM with 10 mL ddH_2_O and filtering through a three-layer lens paper. The conidia were concentrated by 5000 rpm centrifugation at 4 °C for 10 min, resuspended in the centrifuge tubes with 0.2 mL ddH_2_O, and counted using a hemocytometer. The experiment was repeated 3 times with at least 3 replicates each time.

### 4.5. Assay for Conidial Germination and Appressorial Formation

The 200 µL conidia suspensions in 1 × 10^5^/mL were placed on the plastic coverslips and incubated in a humid Petri dish to induce germination and appressorial formation at 28 °C. The samples at a series of time points until 48 h after incubation were examined under an Olympus BX51microscope (Olympus, Japan). The appressorium formation rate was determined as at least 300 conidia for each sample. Cell wall and hyphae septa were visualized by Calcofluor white staining and cellular lipids were strained with Nile-red as described, respectively. The fluorescent samples were detected using a laser confocal fluorescence system Leica SP2 (Leica, Mannheim, Germany).

### 4.6. Pathogenicity Tests

The pathogenicity assay was performed using 14-day-old seedlings of the susceptible rice cultivar CO39. The rice seedlings were spray inoculated with 1 × 10^5^/mL conidia suspensions of all tested strains containing 0.25% (*w*/*v*) gelatin and incubated in a growth chamber at 28 °C and 90% relative humidity for 7–10 days. Lesions were counted from 5-cm leaf tips randomly chosen and the mean densities of the lesions were calculated and statistically compared.

### 4.7. Nucleic Acid Manipulations and Quantitative RT-PCR

Genome DNA was extracted by using the CTAB (hexadecyltrimethylammonium bromide) method [11]. Total RNA extraction followed the method described [11]. Electrophoresis, restricted digestion, ligation reaction and Southern blotting were all carried out by following the standard procedures [42]. For Southern blotting, the genomic DNAs of all tested strains were digested with *Eco*RΙ, separated on 1% agarose, and hybridized using the 2124-bp downstream flanking fragment as a probe.

Total RNA samples isolated from the mycelia, conidia or appressoria were used to synthesize the cDNA using reverse transcriptase. Quantitative RT-PCR was performed on a ABI7500 fast real time PCR system (ABI, Raleigh, NC, USA) with SYBR Premix Ex Taq^TM^ (Takara, Kusatsu, Japan).

### 4.8. Sexual Reproduction

*M. oryzae* strain 2539, Guy11 and mutants, perforated and cross-shaped inocubated on an OMA plate, at 20 °C for 24 h continuous light for 30 days; the morphology of perithecia, ascus and ascospores were observed and photographed. The ascus and ascospores were stained by 0.1% Calcofluor white and 0.1% Nile-red before observation, respectively.

## Figures and Tables

**Table 1:** The strains used in this study.

Strains	Description
Guy11	Wild type
*MoDHX35*-6	Null mutant
*MoDHX35*-7	Null mutant
*MoDHX35*-8	Null mutant
*MoDHX35*-9	Null mutant
*MoDHX35*-5	Random-inserted-transformant
*MoDHX35*-9-10	Complementary transformant
*MoDHX35*-9-16	Complementary transformant

**Table 2:** The primers used in this study.

Primer Name	Primer Sequence
*MoDHX35*-CDS-F1	CGGAATTCTTTTACCTGGTAAAAGTVGGGCGC
*MoDHX35*-CDS-R1	TCCCCCGGGATGGCCGACTTTGATCTGGGTGCA
*MoDHX35*-Com-F1	GCTCTAGAAGCTCAAGCAGACAGACCTAGTTG
*MoDHX35*-Com-R1	TCCCCCGGGGACGATAATGTCAATCTCCGAGGA
*MoDHX35*-Up-F1	GATGGRGCGCCGGATGTGATGAC
*MoDHX35*-Up-R1	GTATTGATTATTTGGGAGGCTTCT
*MoDHX35*-Down-F1	CGGGATCCAAGTACCCTGCGGAAACATC
*MoDHX35*-Down-R1	AAGTTTCTCTCCTCCAAAAAGCTT
*MoDHX35*-Genecheck-F1	CAGTGACAGTTGTCGTTGGA
*MoDHX35*-Genecheck-R1	GTGCTGGATTCTAGTTCTAG
HPH-Check-F1	TGGAGGTCAACACATCAATGCTATT
HPH-Check-R1	CTACTCTATTCCTTTGCCCTCGGAC
Sequence-Up	ACTCGCCGATAGTGGAAACC
*MoDHX35*-Upcheck-F1	CGGTCTGTATTTGGCATACG
Sequence-Down	CCAGTTGCCTAAATGAACCA
*MoDHX35*-Downcheck-R1	TCCTCGTTGTGCAGAGTCTT

## Data Availability

Not applicable.

## Supplementary Materials

The following supporting information can be downloaded at: https://www.mdpi.com/article/10.3390/ijms23169015/s1.
