# Supplementary material for: MoDHX35, a DEAH-Box Protein, Is Required for Appressoria Formation and Full Virulence of the Rice Blast Fungus, Magnaporthe oryzae
Source: Int J Mol Sci. 2022 Aug 12;23(16):9015. doi: 10.3390/ijms23169015 (PMC9409162; doi:10.3390/ijms23169015)
Supplement: Supplementary file 1 [file ijms-23-09015-s001.zip › ijms-1830681-supplementary.pdf]

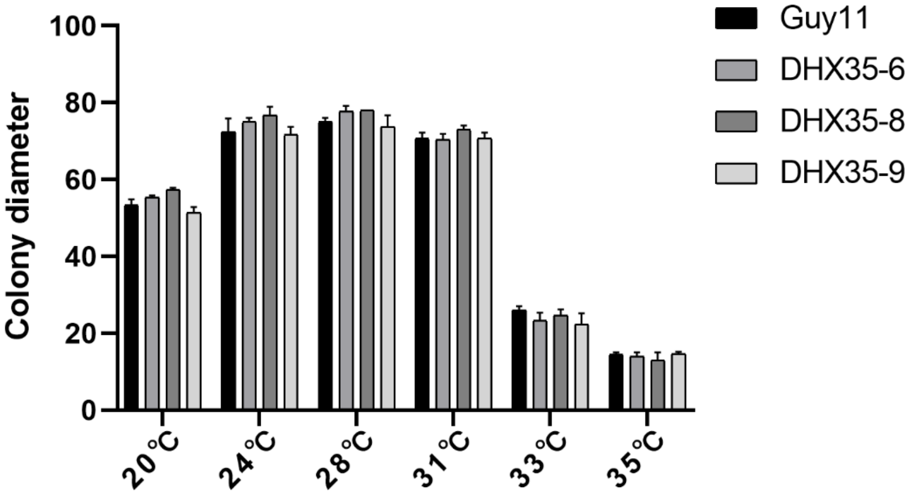

Figure S1. *MoDHX35* mutants are not temperature hypersensitive. The strains were cultured on CM respectively at 20 °C, 24 °C, 28 °C, 31 °C, 33 °C, 35 °C for 9 d, and the colonial diameters were compared.

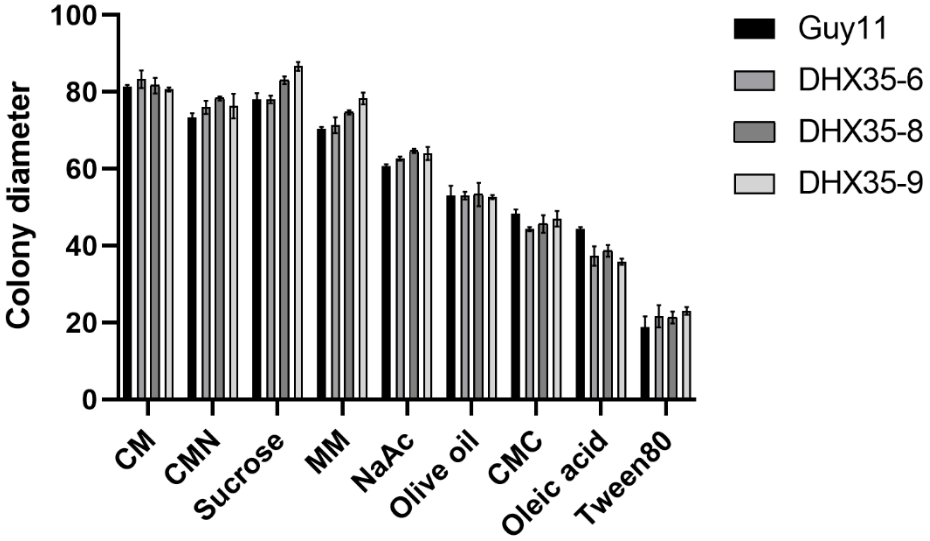

Figure S2. Ability of nutritional utilization was not altered in *MoDHX35* mutants. Media with different Nutrient starvation were used to test whether deletion of *MoDHX35* influenced the nutrient utilization of the fungus. The diameters of the colonies were measured after cultured for 9 d. CM, complete medium; CMC, medium without carbon source; CMN, medium without nitrogen source; MM, Minimal medium; and CMC supplemented with 50 mM sucrose, 1% Tween 80, 50 mM sodium acetate, 1% oleic acid, or 1% olive oil were used.

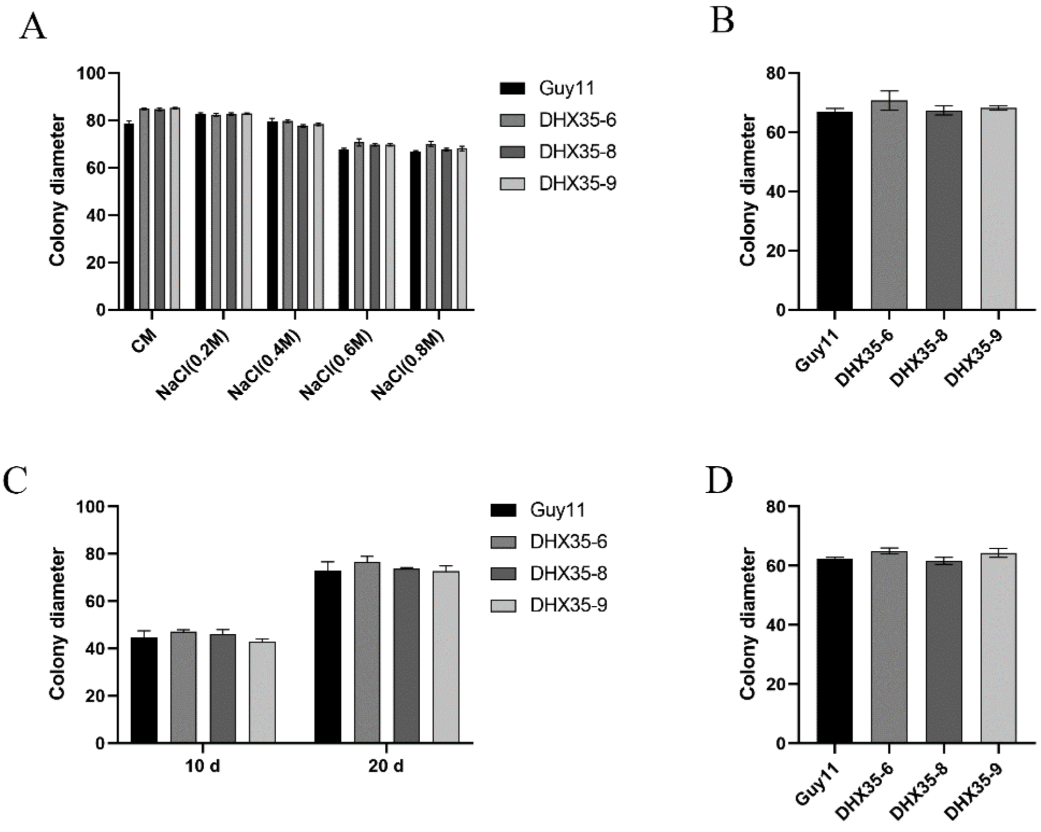

Figure S3. Resistance of *MoDHX35* mutants were not affected to osmotic pressure, Calcofluor white, Cycloheximide and Carbendazim. (A) The strains were cultured on CM supplemented with 0.2 M, 0.4 M, 0.6 M, 0.8 M sodium chloride to test the resistance of the strains to osmotic pressure. The resistance of the mutants to Calcofluor white (B), Cycloheximide (C) and Carbendazim (D).
